# Commentary: RE-AIM Planning and Evaluation Framework: Adapting to New Science and Practice With a 20-Year Review

**DOI:** 10.3389/fpubh.2020.00245

**Published:** 2020-07-03

**Authors:** Kurt C. Stange

**Affiliations:** ^1^Center for Community Health Integration, Research and Development for Community Health and Integrated, Personalized Care, Cleveland, OH, United States; ^2^Center for the Advancement of Primary Health Care for the Public Good Scholar, The Institute for Integrative Health, Cleveland, OH, United States; ^3^Department of Family Medicine and Community Health, Population and Quantitative Health Sciences, Oncology and Sociology, Case Western Reserve University, Cleveland, OH, United States

**Keywords:** RE-AIM framework, interventions, evaluation, program planning, evidence-based

## Introduction

As a boy, Charles Marlow, in Conrad's *Heart of Darkness*, is drawn to explore “blank space of delightful mystery” on the map of Africa. By the time he's old enough to take on the adventure, the blank space now has river names on it, and he's charmed by the uncoiled snake of a river that promises an easy path into what has become “a place of darkness.”

As young investigators, we typically begin with a desire to map, and perhaps to conquer, blank spaces of delightful mystery. We steam boldly up the mighty waterways of well-traveled theory and methods that take us toward the heart of darkness. If we're adventurous, we ignore our advisors' advice and trundle up a tributary. But mostly, we stick to the main channels. We colonize without understanding. Through our colonization, we see the barriers to our dominance, while our theories and methods leave us blind to the motivating delights that await understanding in the murky context.

For more than two decades, the RE-AIM framework (1) has been a bonfire in the decontextualized darkness of health improvement interventions for which rigor is defined by the tenets of internal validity. By providing a framework for also paying attention to external validity, RE-AIM offers a lens for refracting and widening the laser focus of reductionist research to shine light on the multilevel factors that influence health and health care. Its extension to PRISM (2, 3) provides the methods and framework for assessing additional contextual factors essential for researchers who want to illuminate the real world impact of health interventions. Recent applications that use RE-AIM iteratively go even further in trying to make implementation science more rapid and relevant (to stakeholders), and aligned with recognition that context is dynamic, and our models, evaluations and interventions need to evolve as contextualized understanding advances (4).

## An Investigator's Path

The Table 1 on the next page shows two possible trajectories for a junior investigator's pathway—from pilot study to tenure. The path on the left is a tried and true trail to success by focusing on a narrowly-configured problem. It ignores the messiness that is the focus of RE-AIM. The other path, on the right, has more short-term risks. It probably is a bad career move. But is has the potential to make a difference in the wicked problems facing health and health care (5). It considers the multilevel contextual factors addressed by RE-AIM and PRISM.

**Table 1:** Two paths to taking on a complex problem in health or health care.

**Stepping stone along the path**	**Path to short-term career success**	**Path to make a difference for a wicked problem**
Pilot study	Discover *the answer*.	Assess *complex interactions*
Hypothesis	Linear causality	Complex, multilevel causality
Funding	Likely	Unlikely
Intervention	RCT	Adaptive RCTs, interrupted time series…
Assessment	Quantitative	Quantitative & qualitative
Outcome	Decontextualized *answer* proven or disproved!	*Understanding* of complex interacting factors + early insight into what might work in what situation
Dissemination & Implementation	Call for simple dissemination	Call for iterative implementation & shared, contextualized learning
Speaker circuit	Bask in the glory of having *the answer*	Very little audience for a complex answer
Effect on the field	Frustration that things don't replicate, and we don't know why	*Continued learning* of how the system works and what the lever points are in different evolving situations
Next step	Move on to *the next big thing* while the complex problem gets worse	Incremental change until the a tipping point is reached, resulting in *progress on the complex problem*
Tenure	Granted	Denied
Relevant literary quotation	“The horror! The horror!” Joseph Conrad, *Heart of Darkness*	“A mosaic is a conversation with time.” Terry Tempest Williams, *Finding Beauty in a Broken World*

The path to short-term career success is the hero's journey to find ***the answer***. A pilot study identifies a single promising mechanism. The hypothesis of simple, linear cause and effect is easy to explain, and appeals to reviewers and funders. Testing the hypothesis uses the gold standard method of a clinical trial—and all the messy contextual factors are washed from view by the miracle of randomization, allowing the investigator to isolate the effect of a single factor in the rarified group of people willing to leave their intervention choice up to a coin flip. The quantitative assessment of an outcome that can be easily measured leads to simple story of what needs to be implemented and disseminated more widely in the now ***evidence-based intervention***. The possible dead end on this pathway is the dreaded null trial, and the lack of contextual understanding can leave the investigator floundering as to a next step. But a positive trial leads to fame by proposing a simple answer to a complex problem, and the focused research and funding record make an easy case for tenure. It is a while before systematic reviews of dozens of similar trials that have launched other careers lead to the conclusion of “great heterogeneity of treatment effect. More research is needed.” By the time it becomes apparent that the lack of contextual information in such trials makes it impossible to do more than speculate on what that next research should be, the investigator is on to ***the next big*** ***thing***.

The parallel path toward making a difference for a wicked problem attempts at the outset to ***assess complex multilevel causality***. It often involves less easy-to-explain research designs such as interrupted time series, and often integrates quantitative and qualitative methods. This research approach challenges the zeitgeist of rigor as rigid adherence to *a priori* hypotheses—by trying to capture inductive, participatory learning along the way. These studies are challenging to fund because they threaten an easily-ordered worldview. But when interventions show an effect, such studies gather sufficient contextualizing information to be able to do more than speculate on what might work in different situations. And if no treatment effect is identified, their contextualized understanding points the way toward new interventions, iterative implementation, and shared, ***contextualized learning***that represent how knowledge of complex systems actually advances. These kind of results seldom lead to early acclaim, but continued learning of how the system works, and what the lever points are in different evolving situations, leads to incremental change in a learning community, until a tipping point is reached, resulting in real progress on the complex problem.

I have followed both these pathways in my career—brandishing the first path, secreting the second. I mentor many junior investigators who want to do more than look under the lamppost. They want to peer into the heart of darkness of wicked problems. I have to advise them to frame their research around the narrative in the left column of the Table 1, while trying to develop the story on the right. The path on the left is the better career move. It garners the external markers of success that allow the work to continue. The trick in doing this stealth research, is to not be sucked into the illusion that reductionist success is the real goal.

I have managed to be successful by telling the story on the left while living the one on the right. That success has included tenure, many invitations to speak and to influence, sustained funding, appointment as a distinguished university professor, and membership in the National Academy of Medicine. I have been sustained by tremendous colleagues who have played this game together, sustaining each other in the narrative on the right, while living the lie on the left. But I wonder how much more could have been accomplished had we been able to tell the real story all along. I don't want the next generation to have to live the lie, but rather, to overtly track the truth of complex systems.

## Discussion

Our natural human hunger for the simple story makes it challenging to take on the more multifaceted plot line. But RE-AIM and PRISM help us to tell the real story. They shine light on multilevel context from the outset, rather than as an afterthought. Assessing that context means that we can take on the real problems affecting the health and equity of our society, rather than their decontextualized, reductionist shadows.

Let's mainstream the narrative that RE-AIM and PRISM allow us to tell. If we do, we will make much faster progress on what matters.

The alternative? The horror. The horror.

## Author Contributions

The author confirms being the sole contributor of this work and has approved it for publication.

## Conflict of Interest

The author declares that the research was conducted in the absence of any commercial or financial relationships that could be construed as a potential conflict of interest.
